# Effect of Birth Weight and Socioeconomic Status on Children's Growth in Mashhad, Iran

**DOI:** 10.1155/2010/705382

**Published:** 2010-06-23

**Authors:** Ashraf Mohammadzadeh, Ahmadshah Farhat, Rana Amiri, Habibollah Esmaeeli

**Affiliations:** ^1^Medical School and Neonatal Research Center, NICU of Emamreza Hospital, Mashhad University of Medical Science, Iran; ^2^Neonatal Research Center, NICU of Emamreza Hospital, Mashhad University of Medical Science, Iran; ^3^Community Medicine and Public Health Department, Neonatal Research Center, Mashhad University of Medical Science, Iran

## Abstract

*Background*. Socioeconomic status and birth weight are prominent factors for future growing of children. Also Studies show that this criterion is associated with reduced cognitive outcomes, school achievement, and adult work capacity. So in this paper we determined the effects of some socio-economic statuses and birth weight on physical growth of children in Mashhad, Iran. *Method and materials*. This is a cross sectional study that determined effect of socio-economic status and birth weight on weight, heighting and BMI of school age children. Healthy six years old children who were screened before enter, to school were eligible for participating in our study between 6 June 2006 and 31 July. Weight and standing height were documented at birth and measured at 6 years old. Then, their BMI were calculated in childhood period. Data were analyzed by using SPSS software. *Result*. Results show that some socio-economic variables and birth weight is associated with and, perhaps, influence the variation of growth in the children. The variables which show the most consistent and significant association were birth weight, sex, economic status and education of parents. *Conclusion*. In this paper, we found that birth weight, economic status and education parents of neonates have directly significant effect on growth childhood period. We recommended that paying attention to these criteria for improving growth of children in our society should be considered by authorities.

## 1. Introduction

One of the important criteria for healthiness and well-being of children is growth status and growth pattern. The analysis of growth patterns and the detection of aberrant growth patterns provide crucial information for the detection of pathologic condition [1]. So growth and maturation of children is sensitive index of health [2, 3] and is influenced by many factors. However, studies demonstrated that birth weight and social economic status are influenced by growth of children. Growth status is an important marker of health in very low birth weight (VLBW) premature child [4]. The clinical impression is that VLBW children are often under weight and shorter than expected even when corrected for gestational age [1, 3, 5, 6]. There are some studies that evaluated effect of birth weight on growth of children and most of them have reported that birth weight is a significant marker on delayed growth and short stature [7–11]. Socio-economic state (SES) is a concept devised to measure some aspects of education, occupation, and social prestige of a person or a social group [12]. Effects of social factors on the growth rate of children were presented by P founder (1916) for the first time. They observed urban children were taller and grow faster than rural peers [13]. Studies revealed that large number of social-economic variables is associated with the physical development of children. These variables are consisting of parental profession, income, education [14, 15] birth order [16], family size [17], and urbanization [18]. In this study, we determine the effect of birth weight and social-economic status on growth of school age children. Due to the fact that low-birth weight infants do not follow routinely after discharge of NICU in our city besides social-economic status of families, this study will reveal new view about health of children in our society.

## 2. Method

This is a cross-sectional study that examines effect of some social-economic status and birth weight on growth of children. All children have to screen before entering school in our city, so the target population of our study were children who was going to school in Mashhad, Iran between 2005 6 Jan and 2006 31 July and when they referred for screening, we select them randomly in our study. Cluster sample method was used for selection of population study. There are 25 center screening in Mashhad and we selected randomly 10 centers among them. In each 10 center, we choose randomly 240 cases, so totally 2400 new students were entered to our study. Because of the random selection of sites, the sample reflects the basic organizational plan for schools in Mashhad. Design of study was approval by ethical committee of Mashhad University of medical science. Two questionnaires have provided for obtaining social-economic status. At the first questioner, demographic variables such as sex, birth date and nationality were obtained by using birth documentary of children. In the second questioner, social-economic status of children such as economic status, number of children in the family, educational level of the parents, family relationship, home town (urban, rural) and age of parents were obtained by interview with mothers. The socio-economic status questioner was prepared by using some reference [1, 2] and was confirmed by 5 pediatricians. birth weight of children was extracted from vaccination chart while it was prepared for all babies in delivery room. All children in study were checked for height by meter (cm) and weight by digital balance (kg). Then body mas index was determined by using standard growth charts for children 2–14 years of age and calculated as weight in Kilograms/ (height in Meters)^2^ [1]. When growth parameters fall bellow the 5 percentile they are considered as underweight, 5–85 percentile normal weight, and more than 85 percentile overweight. The anthropometric data are presented as mean (SD). For quantitative variables, differences between two groups were tested by using *t*-test and one-way ANOVAs. Categorical variables were analyzed using the chi-square test and fisher exact test when indicated. For controlling confound variables general liner model regression were used. The out-off level of significant was chosen at *P* < .05.

## 3. Result

Of 2400 samples in the study, 180 samples were excluded because of nonavailable of their birth weights. In all samples, 7.91% had birth weight less than 2500 g and others had birth weight more than 2500 g. 52% of children were male and others were female. Also 82% of children had Iranian identify and others were immigrant from Afghanistan, Iraq, or Pakistan. According to the BMI, 14.9% of children were underweight, 73.4% had normal weight and 9.4% overweight. Effect of social-economic variables on weight and height has been shown in Table 1. As shown in Table 1, weight and height has been increased with improved economic status (*P* < .0001). Also result shows that educational level of mothers and fathers had positive effect on weight and height of children (*P* < .001 and *P* < .0001, resp.). People who lived in rural area in had less weight than urban, however height in rural children was less than urban ones but there were not significant difference between them. Weight in mother less than 35 years old was lower than mothers more than 35 years old. So the result indicated that age of mothers also had significant effect weight (*P* = .025). Demographic variables which affect on weight and height of children are shown in Table 2. According to this table, Birth weight also had significant effect on weight and height of children. Children who had birth weight less than 2500 gr had shorter stature and lighter weight than normal ones and there were significant statistical difference between two group (*P* < .01). Effect of socioeconomic status and birth weight on BMI was shown in Tables 3 and 4. Our result shows that most of children in good and medium economic status had normal weight while overweight is most prevalent among low economic status (27.5%). Also children who lived in rural areas were more overweight than in urban area (9.8% versus 5.3%) (Table 3). Percentage of overweight is more prevalent in children with birth weight less than 2500 g (10.3 versus 5) (Table 4). 

## 4. Discussion

Results show that some socio-economic variables and birth weight are associated with and, perhaps, influence the variation of growth in children. The variables which show the most significant association were birth weight, sex, economic status, and educational level of the parents. In our study, maternal and paternal education has significant influence on weight although this effect on height was not significant. Belmont et al. in their study found that maternal education is associated with higher weight and height of children [15]. The other significant factor which was observed in our children was economic status. The result indicated that weight and height of children are directly influenced by economic status. More welfare is a fertile field for growth. Eiben et al. evaluated the effect of socio-economic status on weight and height of children. Their results showed that people of high economic status had more height than low economic status. Also they mentioned that sons of senior salaried employee were taller than unskilled worker about 2.9 cm [16]. Moreover, this study is in accord with other studies that have shown those of lower economic status have lower weight and height [15].Other variable that had significant effect on weight in our study is living place. Our result revealed that people who live in city have more favorite weight than rural ones but effect on height was not significant. The urban environment is thought to be beneficial by providing better education, health care, and availability of food [13]. Although in our study, effect on height was not significant statistically, Peck and V*å*ger*ö* showed that children from larger families tend to be shorter than small families [17]. But some British studies showed that the family size-height relationship is not linear correlation with growth [18]. Silva and et al. determined the effect of socio-economic factors and birth weight on growth of children. Their result revealed that socio-economic status and birth weight were significantly correlated with stature, while the sex of children, maternal age, size of family, and ordinal position of the child in the family were not significantly related to each other [18]. Although most of our samples were in normal range of BMI generally deficiency was revealed more in high risk groups. So finding high risk factors that affect growth of children is valuable parameter. In general, height is a measure of beauty in humans which will affect the self esteem of children in the future. Also weight is mark of heath and deficiency of nutrient substance. In this study, we found that birth weight of neonates has directly significant effect on growth of childhood period. So we can prevent this problem by using remedial policy and follow up LBW children. Also we can predict statue of LBW neonates on the future and it is a finding that needs more investigation. We recommend that teaching parents and society about this hazardous effect is the essential initiative for controlling high risk factors. 

## Figures and Tables

**Table 1:** Socio-economic variables according to weight and height.

Variable	Number (%)	Weight	*P*-value ANOVAs	Height	Number (%)	*P*-value ANOVAs
Economic status						
Good	103 (4.4)	23.55 ± 5.2		119.8 ± 5.6	102 (4.5)	*P* < .001
Medium	1012 (43.6)	21.33 ± 3.86	*P* < .001	117.42 ± 7.2	996 (44)	
Low	1192 (51.4)	20.5 ± 3.13		116.94 ± 5.3	1165 (51.5)	

Education level of mother						
Illiteral or primary	134 (5.8)	20.15 ± 2.82	*P* < .001	117.02 ± 553	135 (5.8)	
Middle school	1420 (61.2)	20.61 ± 3.08		116.79 ± 7.12	1388 (59.6)	*P* = .000
Diploma	551 (23.8)	21.76 ± 4.39		117.97 ± 7.12	620 (26.6)	
Graduated	167 (7.2)	22.85 ± 4.7		118.75 ± 5.6	152 (6.5)	
Postgraduated and doctor	32 (1.4)	27.68 ± 7.66		120.84 ± 5.54	30 (1.2)	

Education of father						
illiteral or primary	148 (6.4)	20.16 ± 2.77	*P* < .001	117.66 ± 5.69	150 (6.5)	
Middle school	1462 (63)	20.56 ± 3.15		117.09 ± 6.9	1450 (63)	*P* = .24
Diploma	568 (24.5)	21.66 ± 4.05		117.13 ± 5.12	570 (24.8)	
Graduated	113 (4.9)	22.2 ± 4.58		117.01 ± 8.7	115 (5)	
Post graduated and doctor	11 (0.5)	25.92 ± 3.64		116.84 ± 5.05	13 (0.5)	

Living place						
Rural	95 (4.1)	20.27 ± 3.67	*P* = .045	116.37 ± 5.64	94 (4)	*P* = .14
Urban	2205 (95.1)	21.044 ± 3.67		117.33 ± 6.29	2163 (94)	

**Table 2:** Effect of birth weight and demographic variables on height and weight.

Variables	Number (%)	Weight	*P*-value	Height	Number (%)	*P*-value
Mother age						
<35 years (1966)	(86) (1944)	20.98 ± 3.64	*P* = .025	117.55 ± 6.1	1512 (67)	*P* = .42
≥35 years (306)	(14) (317)	21.25 ± 3.68		117.18 ± 6.5	749 (33)	

Birth weight						
≤2500 g (*n* = 2080)	2096 (91.6)	21.12 ± 3.09	*P* = .001	117.41 ± 6.5		*P* = .003
<2500 g (*n* = 190)	190 (8.4)	19.8 ± 2.83		115.99 ± 5.2		

Number child						
One child	875 (38)	21.08 ± 3.9		117.8 ± 5.6	875 (39)	
2 child	629 (28)	20.94 ± 3.5	*P* = .327	17.09 ± 6.98	624 (27)	*P* = .25
3 child	341 (15)	21.16 ± 3.49		117.13 ± 5.1	340 (15)	
4 child	211 (9)	20.99 ± 3.3		117.04 ± 8.76	210 (9)	
5 children and more	214 (10)	20.54 ± 3.34		116.84 ± 5.03	213 (10)	

Family relation ship						
No	1592 (70.2)	117.37 ± 6.06	*P* = .347	21.05 ± 3.63	1599 (70.2)	*P* = .333
Yes	674 (29.8)	117.1 ± 6.6		20.89 ± 3.65	678 (29.8)	

Sex						
Male	1201 (53.9)	117.66 ± 6.8	*P* = .003	21.4 ± 3.6	1205 (52.7)	*P* = .000
Female	1024 (46)	116.8 ± 5.51		20.5 ± 3.6	1081 (47.3)	

**Table 3:** Socio-economic variables and birth weight according to BMI.

Variable		BMI		*P*-value
	<5 percentile	5–85 percentile	>85 percentile	
Economic status				
Good	200 (17.2)	810 (75.9)	80 (6.9)	
Medium	134 (13.5)	750 (75.5)	109 (11)	
Low	11 (10.8)	63 (61.8)	28 (27.5)	*P* = .001

Education of mother				
Illiteral or primary	240 (16.8)	115 (80.4)	4 (2.8)	
Middle school	243 (17)	1077 (75.5)	106 (7.4)	
Diploma	654 (11.6)	420 (75.3)	73 (13.1)	*P* = .001
Graduated	121 (10.6)	72 (63.7)	29 (25.7)	
Post graduated and doctor	1 (9.1)	5 (45.5)	5 (45.5)	

Education father				
Illiteral or primary	25 (18.8)	103 (77.4)	5 (3.8)	
Middle school	226 (16.4)	1055 (76.3)	101 (7.3)	
Diploma	74 (13.7)	399 (73.3)	67 (12.4)	
Graduated	19 (11.5)	118 (71.5)	28 (17)	*P* = .001
Post graduated and doctor	2 (6.3)	15 (46.9)	15 (46.9)	

Living place				
Rural	331 (15.4)	1612 (74.8)	12 (9.8)	
Urban	13 (13.8)	76 (80.9)	5 (5.3)	*P* = .001

**Table 4:** Effect of birth weight and Demographic variables on BMI.

Variable		BMI		*P*-value
	<5 percentile	5–85 percentile	>85 percentile	
Mother age				
<35 years (1966)	236 (15.7)	1132 (75.2)	137 (9.1)	
≥35 years (306)	108 (14.4)	555 (74.7)	81 (10.8)	*P* = .357

Birth weight				
≥2500 g (*n* = 2080)	61 (21.6)	207 (73.4)	14 (5)	*P* = .0001
<2500 g (*n* = 190)	285 (14.4)	1492 (75.3)	205 (10.3)	

Number child				
One child	146 (16.8)	635 (73.2)	87 (10)	
2 child	94 (15.1)	465 (74.8)	63 (10.1)	
3 child	44 (13)	262 (77.3)	33 (9.7)	*P* = .64
4 child	30 (14.3)	161 (76.7)	19 (9)	
5 child and more	32 (15.1)	166 (78.31)	14 (6.6)	

Family relationship				
No	247 (15.6)	118 (74.4)	159 (10)	
Yes	96 (14.3)	516 (76.9)	59 (8.8)	*P* = .447

Sex				
Male	116 (9.7)	963 (80.5)	118 (9.9)	
Female	230 (21.5)	739 (69.1)	101 (904)	*P* = .0001
